# BGP-15 Protects against Heart Failure by Enhanced Mitochondrial Biogenesis and Decreased Fibrotic Remodelling in Spontaneously Hypertensive Rats

**DOI:** 10.1155/2021/1250858

**Published:** 2021-01-30

**Authors:** Orsolya Horvath, Katalin Ordog, Kitti Bruszt, Laszlo Deres, Ferenc Gallyas, Balazs Sumegi, Kalman Toth, Robert Halmosi

**Affiliations:** ^1^1st Department of Medicine, University of Pecs, Medical School, Hungary; ^2^Szentágothai Research Centre, University of Pecs, Hungary; ^3^HAS-UP Nuclear-Mitochondrial Interactions Research Group, 1245 Budapest, Hungary; ^4^Department of Biochemistry and Medical Chemistry, University of Pecs, Medical School, Hungary

## Abstract

Heart failure (HF) is a complex clinical syndrome with poor clinical outcomes despite the growing number of therapeutic approaches. It is characterized by interstitial fibrosis, cardiomyocyte hypertrophy, activation of various intracellular signalling pathways, and damage of the mitochondrial network. Mitochondria are responsible for supplying the energy demand of cardiomyocytes; therefore, the damage of the mitochondrial network causes cellular dysfunction and finally leads to cell death. BGP-15, a hydroxylamine derivative, is an insulin-sensitizer molecule and has a wide range of cytoprotective effects in animal as well as in human studies. Our recent work was aimed at examining the effects of BGP-15 in a chronic hypertension-induced heart failure model. 15-month-old male SHRs were used in our experiment. The SHR-Baseline group represented the starting point (*n* = 7). Animals received BGP-15 (SHR-B, *n* = 7) or placebo (SHR-C, *n* = 7) for 18 weeks. WKY rats were used as age-matched normotensive controls (*n* = 7). The heart function was monitored by echocardiography. Histological preparations were made from cardiac tissue. The levels of signalling proteins were determined by Western blot. At the end of the study, systolic and diastolic cardiac function was preserved in the BGP-treated animals. BGP-15 decreased the interstitial collagen deposition via decreasing the activity of TGF*β*/Smad signalling factors and prevented the cardiomyocyte hypertrophy in hypertensive animals. BGP-15 enhanced the prosurvival signalling pathways (Akt/Gsk3*β*). The treatment increased the activity of MKP1 and decreased the activity of p38 and JNK signalling routes. The mitochondrial mass of cardiomyocytes was also increased in BGP-15-treated SHR animals due to the activation of mitochondrial biogenesis. The mitigation of remodelling processes and the preserved systolic cardiac function in hypertension-induced heart failure can be a result—at least partly—of the enhanced mitochondrial biogenesis caused by BGP-15.

## 1. Introduction

Heart failure remained a leading cause of death despite the broadening of therapeutic possibilities [1]. The most important risk factors of heart failure are ischemic heart disease and hypertension [2]. The treatment of hypertension is challenging; there is a high portion of patients who cannot reach the goal blood pressure level having a high risk for the development of heart failure [3]. Sustained elevation of blood pressure induces myocardial remodelling, which is characterized by interstitial fibrosis and cardiomyocyte hypertrophy [4, 5]. These cellular alterations are promoted by oxidative stress [6] and by the activation of various intracellular signal transduction pathways [7, 8]. Numerous studies have demonstrated that mitochondria which are responsible for the cellular energy supply are also damaged in hypertension-induced cardiac remodelling and heart failure [9, 10]. ROS-induced mtDNA damage can be found in the background of these injuries, and mitochondria themselves become the main sources of endogenous ROS production [11]. The long-term presence of these pathophysiological factors finally can lead to heart failure [12]. Spontaneously hypertensive rat (SHR) has become one of the most intensively studied murine strain in experimental cardiology with pathologies resembling human essential hypertension [13, 14]. Therefore, SHR was used in our work as a hypertension-induced heart failure animal model.

BGP-15 (O-[3-piperidino-2-hydroxy-1-propyl]-nicotinic acid amidoxime dihydrochloride) is an insulin sensitizer molecule, with a protective effect in a wide range of experimental models. BGP-15 protects against oxidative stress in ischemia-reperfusion-induced injury in the Langendorff heart perfusion system [15, 16]. Furthermore, it prevents against atrial fibrillation in a transgenic mouse model of heart failure [17]. BGP-15 has beneficial effects on diastolic dysfunction in diabetic cardiomyopathy on Goto–Kakizaki rats [18]. BGP-15 prevented against the imatinib-induced cardiotoxic effects via decreasing the oxidative damages [19]. BGP-15 protects against the ROS-induced mitochondrial ROS production and preserved the mitochondrial membrane potential in the WRL-68 cell line [20]. Nagy et al. demonstrated that BGP-15 protects against the acetaminophen-provoked hepatocellular injury [21]. Moreover, BGP-15 protects lung structure and activates mitochondrial fusion processes in a model of pulmonary arterial hypertension [22].

Fibrotic remodelling, increased ROS production, activation of MAPK signalling pathways, and mitochondrial damage play a significant role in the abovementioned diseases as well as in the pathomechanism of heart failure. Furthermore, it appears that very little information is available or nothing at all on the effect of BGP-15 in the development of hypertensive cardiomyopathy. Therefore, the aim of our study was to investigate the role of BGP-15 in hypertension-induced heart failure.

We focused predominantly on factors that regulate the remodelling processes, myocardial fibrosis, the pattern of related signalling pathways, and the regulation of mitochondrial biogenesis as well.

## 2. Materials and Methods

### 2.1. Ethics Statement

Animals received care according to the Guide for the Care and Use of Laboratory Animals published by the US National Institute of Health (NIH Publication No. 85–23, revised 1996), and the experiment was approved by the Animal Welfare Committee of the University of Pecs, Medical School (permit number: BA02/2000-54/2017).

### 2.2. Experimental Protocol

15-month-old male Wistar Kyoto (WKY) and spontaneously hypertensive rats (Charles River Laboratories, Budapest, Hungary) were used in the experiments. One or two animals were housed per cage under standardized conditions throughout the experiment, with 12 h dark-light cycle in solid-bottomed polypropylene cages, and received commercial rat chew and water ad libitum. Seven SHRs were sacrificed at the beginning of the experiment, as a baseline group (SHR-Baseline). SHRs were randomly divided into two groups: SHR-B and SHR-C. The SHR-B group was treated with BGP-15, a water-soluble compound (25 mg/b.w. in kg/day, *n* = 7), while the SHR-C group received only placebo (*n* = 7, SHR-C) per os for 18 weeks. BGP-15 was a gift from N-Gene Inc. (New York, NY, USA). The dosage of BGP-15 administered in the drinking water was based on preliminary data regarding the volume of daily fluid consumption. WKY rats were used as age-matched normotensive controls (*n* = 7). Noninvasive blood pressure measurements were performed on each animal on three occasions at weeks 0, 9, and 18 of the treatment period. Blood pressure measurements were performed by a noninvasive tail-cuff method as described earlier [23, 24]. Blood pressure was measured by the Non-Invasive Blood Pressure System with rat species platform (Panlab, Harvard Apparatus; LE5002). At the beginning and at the end of the 18-week-long period, echocardiographic measurements were performed. At the end of the 18 weeks, the animals were sacrificed, blood was collected to determine the concentration of plasma brain-derived natriuretic peptide (BNP), then hearts were removed. Atria and great vessels were trimmed from the ventricles, and the weight of the ventricles was measured. Hearts were fixed in 10% formalin for histology or freeze-clamped for Western blot analysis. In order to detect the extent of fibrotic areas, histologic samples were stained with Picrosirius red, and collagen type I immunohistochemistry was made. The phosphorylation state of TGF*β*, Smad2 and 3, Akt-1, GSK-3*β*, and MAPK signalling molecules were monitored by Western blotting. In our research, the following group notations were used according to the applied treatment: WKY: age-matched normotensive Wistar-Kyoto rats; SHR-Baseline: 15-month-old spontaneously hypertensive rats before the treatment period; SHR-C: 19-month-old spontaneously hypertensive rats after the 18-week-long placebo treatment; and SHR-B: 19-month-old spontaneously hypertensive rats after the 18-week-long treatment period with BGP-15.

### 2.3. Echocardiographic Measurements

Transthoracic echocardiography was performed under inhalation anaesthesia at the beginning of the experiment and on the day of sacrifice. The rats were lightly anesthetized with a mixture of 1.5% isoflurane and 98.5% oxygen. The chest of the animals was shaved, and acoustic coupling gel was applied. The animals were imaged in the left lateral position, and a warming pad was used to maintain normothermia. Heart rate did not differ considerably during anaesthesia among the groups. Ventricular dimensions, wall thicknesses, and systolic functions were measured from parasternal short and long-axis views at the midpapillary level. Parameters (E, A, and E') required for the evaluation of diastolic function were measured from the apical 4 chamber view. For the imaging of rats, VEVO 770 high-resolution ultrasound imaging system (VisualSonics, Toronto, Canada) was used, which was equipped with a 25 MHz transducer. The investigators were blinded to the treatment protocol. LV inner dimensions (LVIDd and LVIDs), the thickness of septum and posterior wall (PW), LV end-diastolic volume (LVEDV), LV end-systolic volume (LVESV), E/A, and E/E' ratio were determined. EF (percentage) was calculated by 100 × [(LVEDV − LVESV)/LVEDV].

### 2.4. Determination of Plasma B Type Natriuretic Peptide Level

Blood samples were collected into vacutainer tubes containing EDTA and aprotinin (0.6 IU/ml) and centrifuged at 1600 g for 15 minutes at 4°C to separate plasma, which was collected and kept at −70°C. Plasma B type natriuretic peptide-32 levels (BNP-32) were determined by Enzyme-Linked Immunosorbent Assay method (BNP-32, Rat BNP 32 ELISA Kit, Abcam, ab108815CA, USA) as the datasheet recommends.

### 2.5. Histology

For histological examination, hearts were removed at the end of the study after euthanasia was performed by overdosing isoflurane. Ventricles were fixed in 6% formalin and sliced and embedded in paraffin. Five-micrometer-thick sections were cut serially from the base to the apex by a microtome. Seven animals from each group and 3 sections from each animal were used to determine the degree of cardiac fibrosis. Three images (magnification 10x) were randomly taken from the middle region of the LV wall on each section. The fibrotic area was determined on each image, and the mean value of nine images represents each animal. LV sections were stained with Picrosirius red to detect interstitial fibrosis. Slices were also processed for type I collagen (Bios rabbit polyclonal 1 : 500) immunohistochemistry. The binding was visualized with biotinylated/HRP-conjugated secondary antibody followed by the avidin-biotin-peroxidase detection system (PK-6200 Universal Vectastain ABC Elite Kit, Vector Laboratories, Burlingame, CA) using 3,3′-diaminobenzidine (DAB) as a chromogen. Progress of the immunoreaction was monitored using a light microscope, and the reaction was stopped by the removal of excess DAB with a gentle buffer wash. Animals from each group were used. The degree of fibrosis was quantified by the NIH ImageJ image processing program via its colour deconvolution plug-in.

Picrosirius red staining was performed to measure cardiomyocyte diameter (CD) as a cellular marker of myocardial hypertrophy. Seven animals from each group and 3 sections from each animal were used to determine the cell diameter. Three images (magnification 10x) were randomly taken from the free LV wall on each section. The fitted polygon technique was used to determine the area of the cells. Then, the calculated diameter was used for statistical analysis. In order to evaluate the cardiomyocyte diameter, 250 cardiomyocytes were measured from each animal. The mean value of cell diameter of an animal derived from 250 measurements and each group contained 7 animals.

### 2.6. Western Blot Analysis

Fifty milligrams of heart samples were homogenized in ice-cold Tris buffer (50 mmol/l, pH 8.0) containing protease inhibitor (1 : 100; Sigma-Aldrich Co., #P8340) and phosphatase inhibitor (1 : 100; Sigma-Aldrich Co., #P5726) as well as 50 mM sodium vanadate. The supernatant was harvested in 2x concentrated SDS-polyacrylamide gel electrophoresis sample buffer. Protein levels were measured with NanoDrop. Glyceraldehyde 3-phosphate dehydrogenase (GAPDH; 1 : 1000; Cell Signaling #2118) was used as a loading control. Proteins were separated on 12% SDS-polyacrylamide gel and transferred to nitrocellulose membranes. After blocking (2 h with 5% BSA in Tris-buffered saline contained with 1% Tween-20), membranes were probed overnight at 4°C with primary antibodies recognizing the following antigens: transforming growth factor-*β* (TGF-*β*; 1 : 1000; Cell Signaling #3711), Smad2 (1 : 1000; Invitrogen, 436500), phospho-specific Smad2 Ser465/467 (1 : 1000; Invitrogen, MA5-15122), Smad3 (1 : 1000; Cell Signaling #9523), phospho-specific Smad3 Ser423/425 (1 : 1000; Cell Signaling #9520), protein kinase B (Akt; 1 : 1000; Cell Signaling #9272), phospho-specific Akt-1/protein kinase B-*α* Ser473 (1 : 1000; Cell Signaling #4060), glycogen synthase kinase-3*β* (GSK-3*β*; 1 : 1000; Cell Signaling #9832), phospho-specific glycogen synthase kinase-3*β* Ser9 (1 : 1000; Cell Signaling #5558), p38 mitogen-activated protein kinase (p38MAPK; 1 : 1000; Cell Signaling #8690), phospho-specific p38 mitogen-activated protein kinase Thr180/Tyr182 (1 : 1000; Cell Signaling #4511), c-Jun N-terminal kinase (JNK; 1 : 1000; Cell Signaling #9252), phospho-specific c-Jun N-terminal kinase Thr183/Tyr185 (1 : 1000; Cell Signaling #9255), extracellular signal-regulated kinase (ERK1/2; 1 : 1000; Cell Signaling #4695), phospho-specific extracellular signal-regulated kinase 1/2 Thr202 (1 : 1000; Cell Signaling #4370), mitogen-activated protein kinase phosphatase-1 (MKP-1; 1 : 100; Santa Cruz Biotechnology, sc-373841), peroxisome proliferator-activated receptor gamma coactivator 1-alpha (PGC-1*α*; 1 : 1000; Novus Biologicals, NBP1-04676), cAMP response element-binding protein (CREB; 1 : 1000; Cell Signaling #4820), phospho-specific cAMP response element-binding protein Ser133(1 : 1000; Cell Signaling #9198), 5′ AMP-activated protein kinase (AMPK; 1 : 1000; Cell Signaling #2532), phospho-specific 5′ AMP-activated protein kinase Thr172 (1 : 1000; Cell Signaling #2535), and voltage-dependent anion channel (VDAC; 1 : 1000; Cell Signaling #4661). Membranes were washed six times for 5 min in Tris-buffered saline (pH 7.5) containing 1% Tween-20 (TBST) before the addition of horseradish peroxidase-conjugated secondary antibody (goat antirabbit IgG, Sigma Aldrich Co. A0545, 1 : 3000 dilution; rabbit antimouse IgG, Sigma Aldrich Co., A9044, 1 : 5000 dilution). Membranes were washed six times for 5 min in TBST, and the antibody-antigen complexes were visualized by means of enhanced chemiluminescence. The results of Western blots were quantified using the NIH ImageJ program.

### 2.7. Statistical Analysis

Statistical analysis was performed by SPSS for Windows, version 26.0. All of the data were expressed as the mean ± SEM. The normality of distribution was assessed by the Shapiro-Wilk test. The baseline comparison between the strains was conducted by Student's *t*-test before randomization. The homogeneity of the groups was tested by Levene's test. Differences between treatment groups were determined by one-way ANOVA. For post hoc comparison, Tukey HSD or Dunnett T3 test was applied. A value of *p* < 0.05 was considered statistically significant.

## 3. Results

### 3.1. Effect of BGP-15 Administration on Gravimetric Parameters

At the beginning of the study, the body weight of WKY rats was significantly higher than the SHR rats (WKY: 386.40 ± 4.33 g, SHR-Baseline: 343.21 ± 2.48 g, SHR-C: 346.90 ± 6.65 g, SHR-B: 340.73 ± 6.32 g; *p* < 0.01, WKY vs. SHR groups; Table 1). A similar observation can be made at the end of the study (WKY: 401.45 ± 8.94 g, SHR-C: 358.13 ± 5.08 g, SHR-B: 356.85 ± 4.54 g; *p* < 0.05 WKY vs. SHR groups). At the end of the study, the heart weights (HW) and ventricles weight (VW) were significantly increased in the SHR groups compared to the WKY group (HW: WKY: 1.12 ± 0.04 g, SHR-Baseline: 1.16 ± 0.02, SHR-C: 1.49 ± 0.05 g, SHR-B: 1.23 ± 0.02 g; *p* < 0.01 SHR-B and SHR-C vs. WKY; VW: WKY: 0.95 ± 0.04 g, SHR-Baseline: 1.09 ± 0.02 g, SHR-C: 1.33 ± 0.05 g, SHR-B: 1.23 ± 0.02 g; *p* < 0.01 SHR-C vs. WKY, *p* < 0.01 SHR-B vs. SHR-C). The ratio of ventricular weight to body weight (VW/BW) was increased markedly in the SHR groups compared to WKY animals (VW/BW(mg/g): WKY: 2.28 ± 0.11, SHR-Baseline: 3.19 ± 0.08, SHR-C: 3.73 ± 0.16, SHR-B: 3.21 ± 0.03; *p* < 0.01 SHR groups vs. WKY, *p* < 0.05 SHR-B vs. WKY, *p* < 0.01 WKY vs. SHR-Baseline and SHR-C, *p* < 0.01 SHR-C vs. SHR-Baseline, *p* < 0.01 SHR-B vs. SHR-C). Ventricular weight to the length of right tibia ratio (VW/TL) was also significantly increased (VW/TL (mg/mm): WKY: 21.27 ± 0.79, SHR-Baseline: 24.76 ± 0.82, SHR-C: 29.79 ± 0.94, SHR-B: 25.76 ± 0.46; *p* < 0.05 WKY vs. SHR-Baseline and SHR-B, *p* < 0.01 SHR-C vs. WKY and SHR-Baseline). BGP-15 treatment caused a significant moderation of these ratios (*p* < 0.01 SHR-B vs. SHR-C). The ratio of the lung wet weight-to-dry weight was enhanced in the SHR-C group significantly (lung wet weight/dry weight(g/g): WKY: 4.42 ± 0.26 g/g, SHR-Baseline: 4.51 ± 0.13, SHR-C: 5.68 ± 0.24, SHR-B: 4.68 ± 0.13; *p* < 0.01 SHR-C vs. WKY and SHR-Baseline). BGP-15 caused a significant moderation of this ratio (*p* < 0.01 SHR-B vs. SHR-C).

### 3.2. Effect of BGP-15 Administration on Systolic Blood Pressure and Echocardiographic Parameters

At the beginning of the study, there was a significant difference between the systolic arterial blood pressure of the WKY and the SHR-Baseline group (WKY: 134.85 ± 1.95 mmHg, SHR-Baseline: 214.28 ± 3.70 mmHg; *p* < 0.05; *n* = 7). Systolic arterial blood pressure values did not differ significantly between the SHR groups at the end of the study (SHR-C: 226.14 ± 3.88 mmHg, SHR-B: 216.85 ± 3.90 mmHg; *p* > 0.05; *n* = 7). Long-term BGP-15 treatment apparently did not exert any significant effect on systolic blood pressure.

At the beginning of the study, the septum and posterior wall thickness was significantly higher in SHR animals compared to WKY animals (*p* < 0.01 SHR-Baseline vs. WKY) (Table 1). By the end of the 18-week treatment period, the severity of left ventricular hypertrophy remained unchanged in SHR-C animals. However, wall thicknesses were significantly reduced as a result of BGP-15 treatment (*p* < 0.05, SHR-B vs. SHR-C). LV end-diastolic (LVEDV) and LV end-systolic volumes (LVESV) were also significantly elevated in SHR-C animals (*p* < 0.01, SHR-C vs. WKY, SHR-Baseline). BGP-15 treatment was however able to moderate this elevation in SHR-B animals (*p* < 0.05 vs. SHR-C). LV mass was significantly higher in the SHR-Baseline group compared to WKY (*p* < 0.05 SHR-Baseline vs. WKY). In SHR-C animals, this parameter increased further compared to the initial value (*p* < 0.01, SHR-C vs. WKY; *p* < 0.05, SHR-C vs. SHR-Baseline). This parameter was also decreased in the SHR-B group compared to the nontreated animals (*p* < 0.05, SHR-B vs. SHR-C).

The left ventricular systolic function (EF%) reduced in both the SHR groups compared to the initial value; however, this decrease was more pronounced in the SHR-C group than in the treated animals (*p* < 0.05 SHR-B vs. SHR-C). The diastolic function marker E/E' ratio was significantly increased in the SHR-C group (*p* < 0.05 SHR-C vs. SHR-Baseline), indicating a decrease a diastolic dysfunction.

Meanwhile, the BGP-15 treatment decreased significantly the E/E' ratio in the treated group, compared to the SHR-C animals (*p* < 0.01, SHR-B vs. SHR-C).

### 3.3. Effect of BGP-15 Administration on Plasma BNP Level

By the end of the treatment period, the plasma BNP level increased significantly in the SHR-C group compared to the WKY and SHR-Baseline group (*p* < 0.05, SHR-C vs. WKY and SHR-Baseline group; Table 2). The BGP-15 treatment, however, caused a significant decrease in the level of the biomarker of heart failure in SHR animals (*p* < 0.05, SHR-B vs. SHR-C). The BNP level was only slightly elevated in the SHR-B group.

### 3.4. Effect of the BGP-15 Administration on Interstitial Collagen Deposition

**Figure 1:**
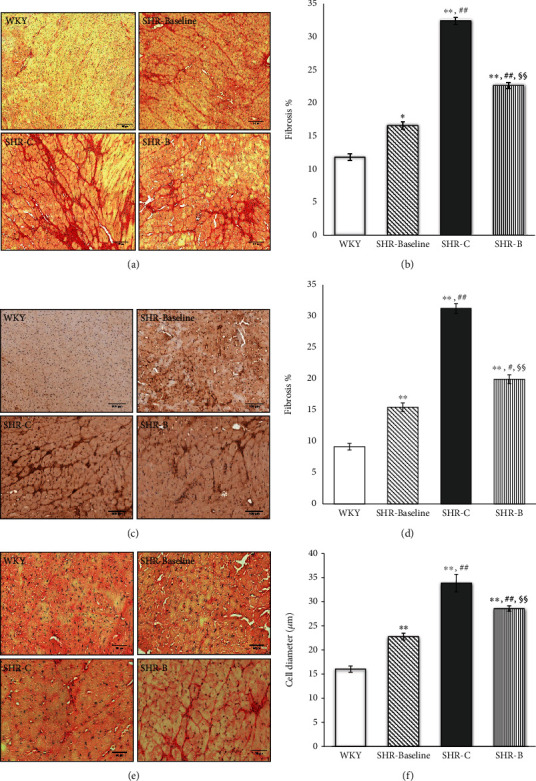
Effect of BGP-15 treatment on the extent of interstitial fibrosis, collagen type I deposition, and on the diameter of cardiomyocytes. Representative histological sections stained with Picrosirius red (a) (*n* = 7). Scale bar: 150 *μ*m, magnification: 10-fold. Densitometric evaluation of the sections is shown (b). ^∗^*p* < 0.05 vs. WKY, ^∗∗^*p* < 0.01 vs. WKY, ^##^*p* < 0.01 vs. SHR-Baseline, ^§§^*p* < 0.01 vs. SHR-C. Representative histological sections detected with collagen type I immunohistochemistry (c) (*n* = 7). Scale bar: 100 *μ*m, magnification: 10-fold. Densitometric evaluation of the sections is shown (d). ^∗∗^*p* < 0.01 vs. WKY, ^##^*p* < 0.01 vs. SHR-C, ^§§^*p* < 0.01 vs. SHR-C. Representative histological sections stained with Picrosirius red (e) (*n* = 7). Scale bar: 50 *μ*m, magnification: 10-fold. The average cellular diameter in the different groups is shown (f). ^∗∗^*p* < 0.01 vs. WKY, ^#^*p* < 0.05 vs. SHR-Baseline, ^§§^*p* < 0.01 vs. SHR-C. WKY: age-matched normotensive Wistar-Kyoto rats; SHR-Baseline: 15-month-old spontaneously hypertensive rats; SHR-C: 19-month-old spontaneously hypertensive rats received placebo for 18 weeks; SHR-B: 19-month-old spontaneously hypertensive rats received BGP-15 for 18 weeks.

**Figure 2:**
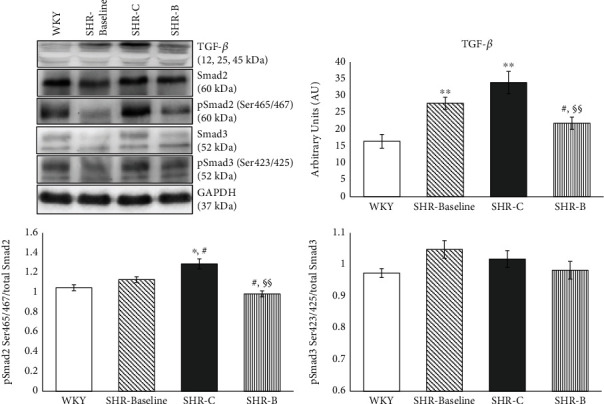
Effect of BGP-15 treatment on the TGF*β*/Smad signalling pathway. Representative Western blot analysis of TGF*β*, Smad2, Smad3, and phosphorylation and densitometric evaluation are shown. GAPDH was used as a loading control. WKY: age-matched normotensive Wistar-Kyoto rats, *n* = 7; SHR-Baseline: 15-month-old spontaneously hypertensive rats, *n* = 7; SHR-C: nontreated spontaneously hypertensive rats, *n* = 7; SHR-B: spontaneously hypertensive rats receiving BGP-15 for 18 weeks, *n* = 7. Values are mean ± SEM. ^∗^*p* < 0.05 vs. WKY, ^∗∗^*p* < 0.01 vs. WKY, ^#^*p* < 0.05 vs. SHR-Baseline, ^§§^*p* < 0.01 vs. SHR-C.

Histological staining of the left ventricle of the heart was performed with Picrosirius red staining (Figures 1(a) and (b)) and collagen I immunohistochemistry (Figures 1(c) and (d)), which was used to monitor the degree of fibrosis. Only a low amount of interstitial collagen could be seen in the WKY group with Picrosirius red staining (Figure 1(a)). The extent of fibrosis was significantly higher in the SHR groups compared to the WKY group (*p* < 0.05, SHR-Baseline vs. WKY; *p* < 0.01, SHR-C and SHR-B vs. WKY; Figure 1(a)). Chronic high blood pressure-induced heart failure caused a further elevation of collagen deposition in the SHR-C group (*p* < 0.01, vs. SHR-Baseline group). The BGP-15 treatment however resulted in a significant decrease in the amount of interstitial fibrosis in the SHR-B group compared to nontreated hypertensive animals (*p* < 0.01, SHR-B vs. SHR-C; Figure 1(a)) (WKY: 11.78 ± 1.00%; SHR-Baseline: 16.59 ± 1.03%; SHR − C : 32.42 ± 1.52%; SHR − B : 22.64 ± 1.09%; Figure 2(b)).

Similar observations were made in the case of type I collagen immunohistochemistry (Figures 1(c) and (d)). Moderate interstitial collagen deposition was observed in the WKY group (WKY: 9.15 ± 0.54%; SHR-Baseline: 15.45 ± 0.69%; SHR-C: 31.24 ± 0.77%; SHR-B: 19.92 ± 0.72%; Figure 1(d)), in the case of hypertensive groups, even the initial value was higher than the in the WKY group (*p* < 0.01 vs. SHR-Baseline). This elevation became more pronounced by the end of the treatment period (*p* < 0.01 SHR-C vs. WKY, SHR-Baseline groups; Figure 1(c)). Due to the treatment, the interstitial collagen deposition was significantly decreased in the SHR-B group compared to the SHR-C group (*p* < 0.01; Figure 1(c)). It can be concluded that BGP-15 treatment significantly reduced the formation of fibrotic deposits in the myocardium (Figures 1(a)–(d)).

### 3.5. Effect of BGP-15 Administration on the Diameter of Cardiomyocytes

Histological sections from the left ventricle of the heart stained with Picrosirius red were also used to study the cell diameters (Figures 1(e) and (f)). The diameter of cardiomyocytes was markedly elevated in SHR groups compared to the WKY group (WKY: 16.02 ± 0.64 *μ*m; SHR-Baseline: 22.76 ± 0.70 *μ*m; SHR-C: 33.86 ± 1.82 *μ*m; SHR-B: 28.57 ± 0.57 *μ*m; Figure 1(f)). This difference was the most pronounced in the case of SHR-C (*p* < 0.01, SHR-C vs. WKY). The BGP-15 treatment resulted in significantly lower cell diameters in the SHR-B group compared to the SHR-C group (*p* < 0.01; SHR-B vs. SHR-C; Figure 1(e)).

### 3.6. Effect of BGP-15 Administration on the TGF-*β*/SMAD Signalling Pathway

The level of TGF-*β* was significantly elevated in all hypertensive groups compared to the WKY group (*p* < 0.05, SHR-B vs. WKY, *p* < 0.01 SHR-Baseline, SHR-C vs. WKY; Figure 2). In the case of the SHR-C group, a further increasing tendency could be seen by the end of the treatment period compared to the baseline values (NS). However, the BGP-15 treatment caused a significant decrease in the TGF-*β* level compared to the untreated SHR animals (*p* < 0.01, SHR-B vs. SHR-C); moreover, the TGF-*β* level in this group was even lower than in the SHR-Baseline group (*p* < 0.05, SHR-B vs. SHR-Baseline; Figure 2). In the case of Smad2 phosphorylation, we observed a mild increase in the SHR-Baseline compared to the WKY; however, this elevation was not significant. The phosphorylation of Smad2^Ser465/467^ was significantly increased in the SHR-C compared to the WKY and Baseline groups (*p* < 0.05). BGP-15 treatment resulted in a significant reduction in the phosphorylation level of Smad2^Ser465/467^ in the treated group (*p* < 0.01 SHR-B vs. SHR-C). There were no significant differences regarding the phosphorylation of Smad3^Ser423/425^ between the groups (Figure 2). GAPDH was used as a loading control.

### 3.7. Effect of BGP-15 Administration on the Phosphorylation Level of Akt-1 and GSK-3*β*

**Figure 3:**
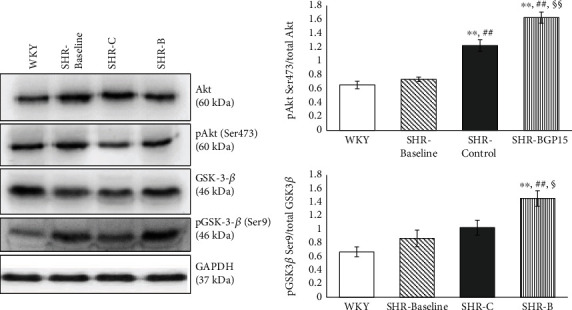
Effect of BGP-15 treatment on the phosphorylation of Akt-1^Ser473^ and GSK-3*β*^Ser9^. Representative Western blot analysis of Akt-1 and GSK-3*β* phosphorylation and densitometric evaluation are shown. GAPDH was used as a loading control. WKY: age-matched normotensive Wistar-Kyoto rats, *n* = 7; SHR-Baseline: 15-month-old spontaneously hypertensive rats, *n* = 7; SHR-C: nontreated spontaneously hypertensive rats, *n* = 7; SHR-B: spontaneously hypertensive rats receiving BGP-15 for 18 weeks, *n* = 7. Values are mean ± SEM. ^∗∗^*p* < 0.01 vs. WKY, ^##^*p* < 0.01 vs. SHR-Baseline, ^§^*p* < 0.05 vs. SHR-C, ^§§^*p* < 0.01 vs. SHR-C.

The level of Akt-1^Ser473^ phosphorylation was moderate in the WKY group as well as in the SHR-Baseline group (Figure 3.). In the SHR-C group, the phosphorylation of Akt-1^Ser473^ was increased slightly, but significantly (*p* < 0.01 SHR-C vs. WKY and SHR-Baseline groups; Figure 3). However, BGP-15 treatment increased a marked increase in the Akt-1^Ser473^ phosphorylation in SHR-B animals (*p* < 0.01 SHR-B vs. SHR-C group).

The phosphorylation level of GSK-3*β*^Ser9^ was low in the WKY group similar to the Akt-1^Ser473^ phosphorylation. In the SHR-Baseline and the SHR-C groups, however, slightly but not significantly elevated phosphorylation could be seen. The highest phosphorylation of GSK-3*β*^Ser9^ was measured in the SHR-B group. This elevation was highly significant to other SHR groups (*p* < 0.05 SHR-B vs. SHR-C; *p* < 0.01, SHR-B vs. SHR-Baseline group; Figure 3). GAPDH was used as a loading control.

### 3.8. Effect of BGP-15 Administration on the Activity of MAPKs

**Figure 4:**
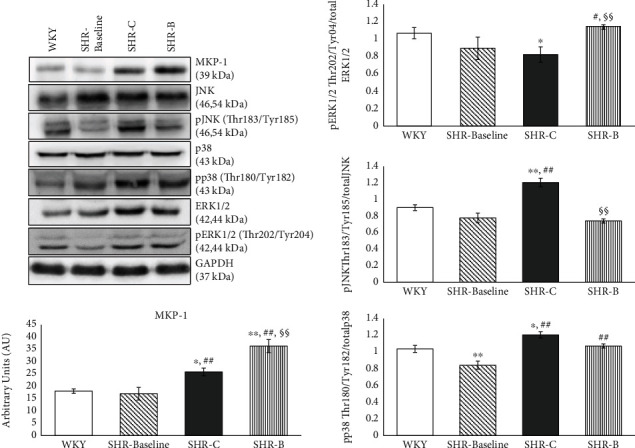
Effect of the BGP-15 treatment on the phosphorylation state of MAP kinases and on MKP-1. Representative Western blot analysis of MKP-1 as well as ERK1/2, p38, and JNK phosphorylation. Densitometric evaluation is also shown. GAPDH was used as a loading control. WKY: age-matched normotensive Wistar-Kyoto rats, *n* = 7; SHR-Baseline: 15-month-old spontaneously hypertensive rats, *n* = 7; SHR-C: nontreated spontaneously hypertensive rats, *n* = 7; SHR-B: spontaneously hypertensive rats receiving BGP-15 for 18 weeks, *n* = 7. Values are mean ± SEM. ^∗^*p* < 0.05 vs. WKY, ^∗∗^*p* < 0.01 vs. WKY, ^##^*p* < 0.01 vs. SHR-Baseline, ^§§^*p* < 0.01 vs. SHR-C.

The level of MKP-1 protein was low in the WKY and SHR-Baseline groups (Figure 4). A significant increase was however observed in the SHR-C group (*p* < 0.01, SHR-C vs. WKY as well as SHR-Baseline groups). The amount of MKP-1 protein increased further in the SHR-B group as a result of the BGP-15 treatment (*p* < 0.01, SHR-B vs. SHR-C; Figure 4). The level of Erk1/2^Thr202/Tyr204^ phosphorylation was less pronounced in the SHR-C group compared to the WKY group and to baseline level (*p* < 0.05, SHR-C vs. WKY, Figure 4). BGP-15 treatment however caused a significant elevation in the phosphorylation of Erk1/2^Thr202/Tyr204^ compared to the SHR-C group (*p* < 0.01, SHR-B vs. SHR-C; Figure 4). The level of p38-MAPK^Thr180/Tyr182^ and JNK^Thr183/Tyr185^ phosphorylation was low in the WKY and in the SHR-Baseline groups (Figure 4). The highest phosphorylation level of p38-MAPK^Thr180/Tyr182^ and JNK^Thr183/Tyr185^ could be seen in the SHR-C animals (*p* < 0.01, SHR-C vs. WKY and SHR-Baseline). The BGP-15 treatment reduced this phosphorylation of p38-MAPK^Thr180/Tyr182^ and JNK^Thr183/Tyr185^ too, and this reduction was significant in the case of JNK^Thr183/Tyr185^ compared to the SHR-C group (*p* < 0.01; Figure 4). GAPDH was used as a loading control.

### 3.9. Effect of BGP-15 Administration on the Regulation of Mitochondrial Biogenesis

**Figure 5:**
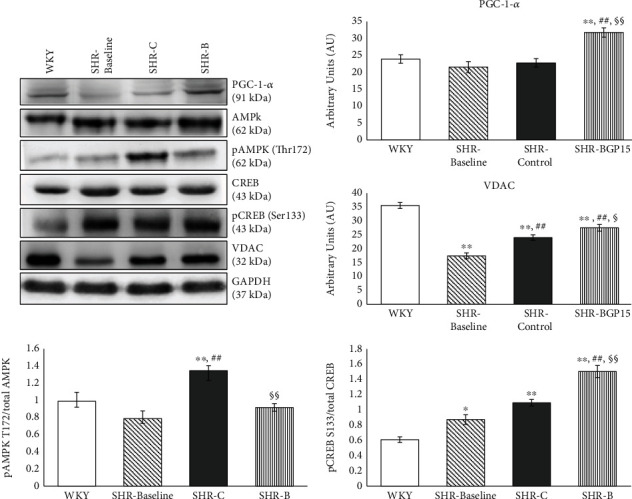
Effect of the BGP-15 treatment on the regulation of mitochondrial biogenesis. Representative Western blot analysis of PGC-1*α*, VDAC, CREB, AMPK, and phosphorylation of CREB and AMPK. Densitometric evaluation is also shown. GAPDH was used as a loading control. WKY: age-matched normotensive Wistar-Kyoto rats, *n* = 7; SHR-Baseline: 15-month-old spontaneously hypertensive rats, *n* = 7; SHR-C: nontreated spontaneously hypertensive rats, *n* = 7; SHR-B: spontaneously hypertensive rats receiving BGP-15 for 18 weeks, *n* = 7. Values are mean ± SEM. ^∗^*p* < 0.05 vs. WKY, ^∗∗^*p* < 0.01 vs. WKY, ^##^*p* < 0.01 vs. SHR-Baseline, ^§^*p* < 0.05 vs. SHR-C, ^§§^*p* < 0.01 vs. SHR-C.

There were no significant differences between the WKY, SHR-Baseline, and SHR-C groups regarding the PGC-1*α* level (Figure 5). However, the BGP-15 treatment caused a significant increase in the amount of PGC-1*α* compared to the nontreated hypertensive animals (*p* < 0.01, SHR-B vs. SHR-Baseline and SHR-C group; Figure 5). In the case of AMPK^Thr172^ phosphorylation, a significant increase was observed in the SHR-C group compared to the SHR-Baseline group (*p* < 0.01, SHR-C vs. SHR-Baseline; Figure 5). The BGP-15 treatment significantly reduced the phosphorylation of AMPK^Thr172^ compared to the SHR-C group (*p* < 0.01 SHR-B vs. SHR-C). The CREB^Ser133^ phosphorylation was modest in the WKY group similar to the phosphorylation of AMPK^Thr172^ (Figure 5). There was a significant increase in the phosphorylation level of CREB^Ser133^ in the SHR-C group compared to the baseline value and to the normotensive animals (*p* < 0.01, SHR-C vs. WKY; Figure 5). However, the BGP-15 treatment caused a further increase in the CREB^Ser133^ phosphorylation compared to nontreated SHR animals (*p* < 0.05 SHR-B vs. SHR-C group) and to the baseline value (*p* < 0.01 SHR-B vs. SHR-Baseline). The highest VDAC protein level was observed in the WKY group. This level was significantly lower in the hypertensive groups (*p* < 0.01 WKY vs. SHR-Baseline, SHR-C, and SHR-B). By the end of the treatment period, VDAC became higher compared to the initial value (*p* < 0.01, SHR-C vs. SHR-Baseline). A further significant increase was seen in the SHR-B group (*p* < 0.05 SHR-B vs. SHR-C). GAPDH was used as a loading control.

## 4. Discussion

In this work, we aimed to examine the cardioprotective effect of BGP-15 in chronic hypertension-induced heart failure. The major findings of this study are that BGP-15 has a positive effect on cardiac function and on remodelling processes by inhibiting profibrotic signalling factors and by promoting mitochondrial biogenesis in an animal model of hypertension-induced heart failure.

SHR was used to provoke hypertension-induced heart failure. SHR is a widely used animal model in experimental cardiology because it resembles the human essential hypertension [13]. 15-months-old SHRs already showed the unquestionable signs of hypertensive heart disease at the start of the experiments. Left ventricular wall thicknesses and the LV mass were markedly increased in the SHR animals compared to normotensives (Table 2). However systolic left ventricular function was still normal in both normotensive and hypertensive animals. This is in accordance with the results of other workgroups and with our former results [25, 26]. The signs of left ventricular hypertrophy remained marked also by the end of the study in SHR animals. However, systolic (EF%) as well as diastolic left ventricular function (E/E') worsened significantly by that time, and animals showed the signs of heart failure. The worsening of these parameters was considerably lower due to the BGP-15 treatment of hypertensive animals (Table 2). This result supports and complements the results of Sapra et al. that BGP-15 has beneficial effects on cardiac function in murine heart failure [17]. Moreover, left ventricular hypertrophy as well as the severity of left ventricular diastolic dysfunction was not only moderated due to BGP-15 treatment but also improved slightly, showing a so-called reverse remodelling phenomenon.

The BNP plasma level is a biomarker of heart failure. There is a direct proportionality between the severity of heart failure and the BNP level [27]. The marked increase of BNP that was seen in nontreated hypertensive animals (SHR-C) was also positively affected by BGP-15, because it decreased the BNP level to the level of normotensive animals (Table 2).

Hypertensive heart disease including heart failure is characterised by cardiac fibrosis. Extracellular matrix (ECM) remodelling can be observed during cardiac fibrosis, which leads to abnormalities in matrix composition and quality, as well as to decrease heart function [4, 28]. Heart failure is characterized by an increased collagen type I deposition. Thus, collagen type I is a marker of cardiac fibrosis. Collagen type I is one of the major components of the adult human cardiac tissue (approximately 85%) while collagen type III is the other important component (11%) [29]. Ventricular remodelling was characterized by cardiomyocyte hypertrophy and an extensive myocardial collagen deposition [30]. Both phenomena could be seen in hypertensive animals. BGP-15, however, prevented against hypertension-induced cardiac interstitial fibrosis and cardiomyocyte hypertrophy (Figure 1). The transforming growth factor-*β* (TGF*β*)/Smad signalling route has a major role in the regulation of cardiac fibrosis [31, 32]. Activation of TGF-*β*/Smad signalling promotes myofibroblast formation and extracellular matrix (ECM) production that are leading to cardiac fibrosis [33]. In our recent work, hypertension induced a marked cardiac fibrosis by the activation of the TGF-*β*/Smad pathway (Figure 2). Both the level of TGF-*β* and the phosphorylation of Smad2^Ser465/467^ were significantly reduced due to BGP-15 treatment; therefore, it can be a mechanism in the background of decreased fibrosis, observed in the SHR-B group. The BGP-15-induced inhibition of fibrosis and cardiomyocyte hypertrophy are on the other hand the main causes of the improved cardiac function and structure compared to nontreated SHRs (Tables 1 and 2).

It is well known that the MAPK signalling pathway also plays an important role in the pathogenesis of hypertension-induced cardiac remodelling and heart failure [7, 34, 35]. MAP kinases, predominantly p38 MAPK and JNK, are other important regulators of myocardial fibrosis [24, 36, 37]. The activity of MAP kinases are regulated by dual-specificity phosphatases (DUSPs) or MAPK phosphatases (MKPs) that can dephosphorylate MAPKs and in this way regulate—actually inhibit—their activity [34, 38]. In our recent work, the expression of MKP-1 increased significantly due to BGP-15 treatment in comparison with the SHR-C animals (Figure 4) As a consequence of the increased amount of MKP-1, the p38 MAPK and JNK phosphorylation decreased in the treated animals, in accordance with several previous studies that also confirmed the beneficial effect of BGP-15 on the phosphorylation state of p38 MAPK and JNK (Figure 4) [19, 22]. In the case of ERK phosphorylation, an opposite change could be seen in our work, because BGP-15 increased the ERK1/2 phosphorylation (Figure 4). Regarding BGP-15, there are studies that are in accordance with our results. Szabo et al. demonstrated that BGP-15 treatment increased the phosphorylation of ERK1/2 in WRL-68 cells [22]. However, in another work, BGP-15 decreased the phosphorylation of ERK1/2 in imatinib-induced cardiotoxicity [19]. Because ERK1/2 is a member of prosurvival signalling factors, its activation is beneficial in the failed myocardium [25, 39].

Akt-1 also belongs to prosurvival signalling factors, and it can promote “physiological” hypertrophy; however, it inhibits the pathological hypertrophy that is mainly characterised by cardiac collagen accumulation [40–42]. GSK-3*β* is a downstream target of Akt-1 and Akt-1 which via the phosphorylation of GSK-3*β* can promote the survival of chronically stressed cardiomyocytes in heart failure as demonstrated by previous works [25]. The cytoprotective effect due to increased phosphorylation of Akt-1 and GSK-3*β* is mediated via their protective effect on the structure and function of mitochondria [43]. In our recent study, BGP-15 increased significantly the phosphorylation of Akt-1 and GSK-3*β* compared to the nontreated SHR animals (Figure 3); therefore, the BGP-15 treatment activates the prosurvival signalling pathways.

The contractile function of cardiomyocytes is in strong correlation with the energy-producing capacity of the mitochondrial network [44]. Numerous studies have demonstrated that mitochondrial biogenesis is an essential step in mitochondrial quality control and is a highly vulnerable process in heart failure [45, 46]. PGC-1*α* is the master signalling factor of biogenesis, and it is regulated in different ways, among others by AMPK and CREB [47–49]. We found that the expression level of PCG-1*α* increased due to BGP-15 treatment compared to SHR-C animals (Figure 5). Phosphorylation of AMPK was however reduced as a result of the treatment (Figure 5). AMPK activation is a consequence of increased AMP to ATP ratio, which is a sign of energy depletion. Therefore, this reduction of AMPK phosphorylation indicates a favourable change in the energy production of cardiomyocytes [50, 51]. Phosphorylation of CREB was on the other hand increased in BGP-15-treated SHR animals compared to nontreated ones (Figure 5). BGP-15 via the activation of CREB increased the expression level of PGC-1*α*, which in turn can yield in enhanced mitochondrial biogenesis and in increased high energy phosphate production. CREB transcription factor can also increase the production of MKP-1 and thereby can decrease the activity of the MAPK signal pathway, too [52, 53], which could be seen in our study. We determined the amount of VDAC, an outer mitochondrial membrane protein to characterize the number of mitochondria in cardiomyocytes. The elevation of VDAC in BGP-15-treated animals proved that there is an increased mitochondrial biogenesis and mitochondrial mass in cardiomyocytes.

**Figure 6:**
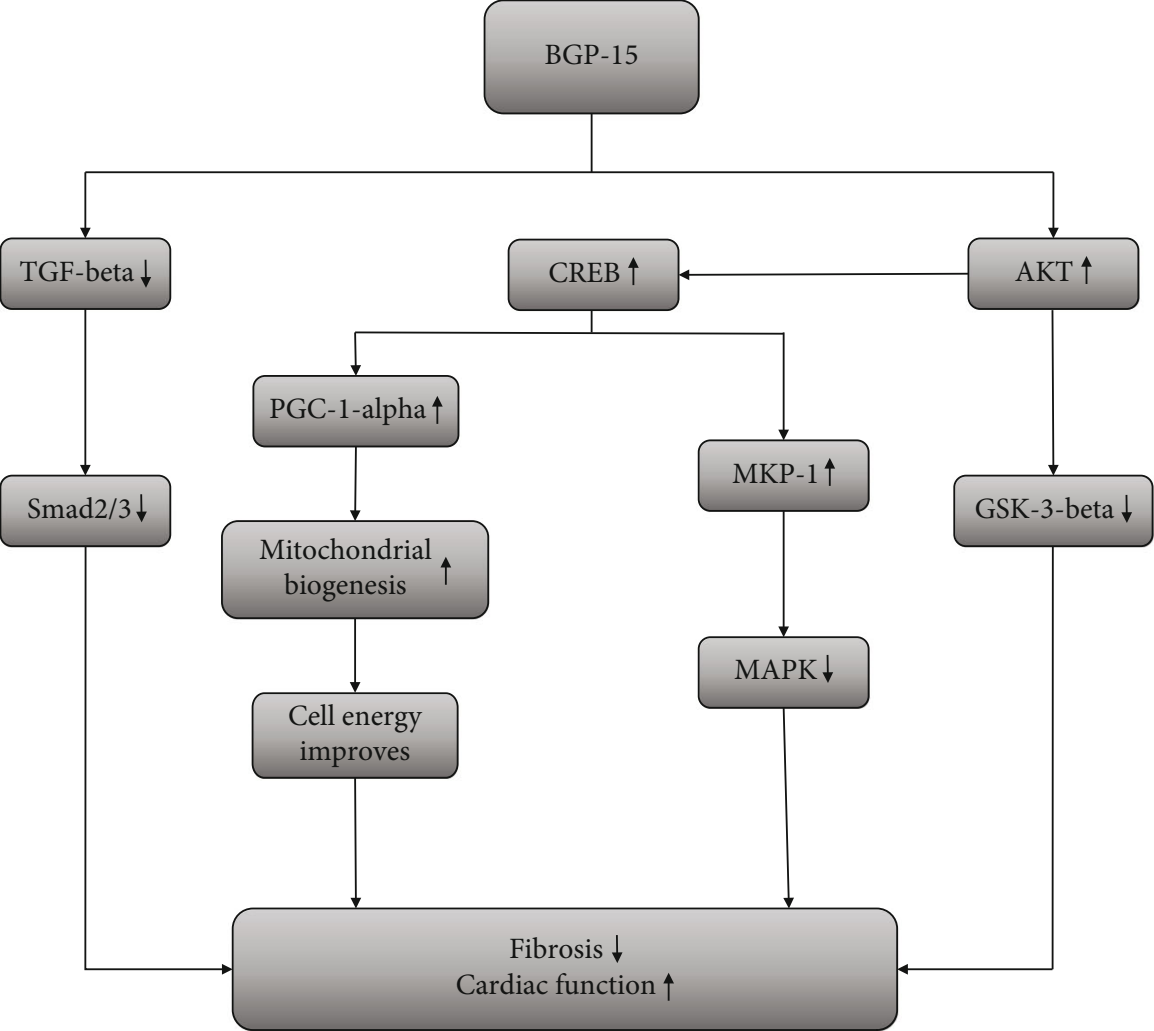
The suspected mechanism of BGP-15 treatment in a hypertension-induced heart failure model. BGP-15 has a beneficial effect against hypertension-induced cardiac remodelling and cardiac fibrosis. The BGP-15 treatment decreases the activity of TGF*β*/Smad and MAPK signalling factors and in this way prevents against hypertension-induced interstitial collagen deposition. BGP-15 favourably influences the prosurvival signalling pathways. Moreover, the mitochondrial biogenesis is activated due to BGP-15 administration, thereby resulting in an increase in mitochondrial mass.

In conclusion, BGP-15 treatment exerted a marked protective effect against the development of hypertension-induced heart failure via the inhibition of the fibrotic remodelling of the heart. This effect could be explained by its beneficial effect on signal transduction factors and by the increased mitochondrial biogenesis (Figure 6).

## Figures and Tables

**Table 1:** Effect of BGP-15 administration on gravimetric parameters of SHR animals.

	WKY (*n* = 7)	SHR-baseline (*n* = 7)	SHR-C (*n* = 7)	SHR-B (*n* = 7)
BW^START^ (g)	386.40 ± 4.33	343.21 ± 2.48^∗∗^	346.90 ± 6.65^∗∗^	340.73 ± 6.32^∗∗^
BW^END^ (g)	401.45 ± 8.94	—	358.13 ± 5.08^∗∗^	356.85 ± 4.54^∗∗^
HW^END^ (g)	1.12 ± 0.04	1.16 ± 0.02	1.49 ± 0.05^∗∗^^,##^	1.23 ± 0.02^§§^
VW^END^ (g)	0.95 ± 0.04	1.09 ± 0.02	1.33 ± 0.05^∗∗^^,##^	1.14 ± 0.02^§§^
VW/BW^END^ (mg/g)	2.28 ± 0.11	3.19 ± 0.08^∗∗^	3.73 ± 0.16^∗∗^^,##^	3.21 ± 0.03^∗^^,§^
VW/TL^END^ (mg/mm)	21.27 ± 0.79	24.76 ± 0.82^∗^	29.79 ± 0.94^∗∗^^,##^	25.76 ± 0.46^∗^^,§§^
Lung wet weight/weight^END^ (g/g)	4.42 ± 0.26	4.51 ± 0.13	5.68 ± 0.24^∗∗^^,##^	4.68 ± 0.13^§§^

BW^START^: body weight at the beginning of the treatment; BW^END^: body weight at the end of the treatment; HW^END^: heart weight at the end of the treatment; VW^END^: ventricles weight at the end of the treatment; TL^END^: length of the right tibia at the end of the treatment. Values are means + SEM. WKY: age-matched normotensive Wistar-Kyoto rats, *n* = 7; SHR-Baseline: 15-month-old spontaneously hypertensive rats, *n* = 7; SHR-C: nontreated spontaneously hypertensive rats, *n* = 7; SHR-B: spontaneously hypertensive rats receiving BGP-15 for 18 weeks, *n* = 7. ^∗^*p* < 0.05 vs. WKY, ^∗∗^*p* < 0.01 vs. WKY, ^##^*p* < 0.01 vs. SHR-Baseline, ^§^*p* < 0.05 vs. SHR-C, ^§§^*p* < 0.01 vs. SHR-C.

**Table 2:** Effect of BGP-15 treatment on echocardiographic parameters.

	WKY (*n* = 7)	SHR-Baseline (*n* = 7)	SHR-C (*n* = 7)	SHR-B (*n* = 7)
	Mean ± SEM	Mean ± SEM	Mean ± SEM	Mean ± SEM
Septum	1.93 ± 0.03	2.29 ± 0.07^∗∗^	2.32 ± 0.07^∗∗^	2.09 ± 0.08^∗^^,§^
PW	1.90 ± 0.04	2.06 ± 0.06^∗^	1.97 ± 0.08^∗^	1.81 ± 0.07^§^
LVIDd (mm)	7.61 ± 0.14	7.75 ± 0.15	8.55 ± 0.23^∗∗^^,##^	8.31 ± 0.18^∗∗^
LVIDs (mm)	4.54 ± 0.13	4.60 ± 0.21	5.87 ± 0.31^∗∗^^,##^	5.19 ± 0.32^§^
LVEDV (*μ*l)	310.25 ± 12.85	323.07 ± 14.59	402.40 ± 24.76^∗∗^^,##^	377.19 ± 17.37^∗∗^
LVESV (*μ*l)	96.01 ± 6.85	101.51 ± 12.27	175.52 ± 22.46^∗∗^^,##^	137.23 ± 16.46^∗∗^^,§^
LV mass (mg)	1029.81 ± 43.84	1384.42 ± 40.69^∗∗^	1587.38 ± 106.36^∗∗^^,#^	1321.44 ± 75.58^∗^^,§^
EF%	70.48 ± 1.12	69.59 ± 2.41	57.21 ± 3.02^∗∗^^,##^	64.30 ± 2.88^∗^^,§^
E/A	1.37 ± 0.07	1.70 ± 0.09^∗∗^	2.02 ± 0.06^∗∗^	1.27 ± 0.08^§§^
E/E'	30.45 ± 2.00	30.32 ± 2.98	40.41 ± 2.94^∗∗^^,##^	25.71 ± 3.03^§§^
BNP (pg/ml)	302.76 ± 13.76	325.19 ± 10.89	755.14 ± 33.34^∗^^,#^	352.04 ± 22.50^§^

Septum: thickness of the septum; PW: thickness of the posterior wall; LVIDd: left ventricular (LV) inner diameter end-diastolic; LVIDs: LV inner diameter end-systolic; LVEDV: LV end-diastolic volume; LVESV: LV end-systolic volume; LV mass: calculated weight of left ventricle; EF: ejection fraction; E: mitral peak velocity of early filling; A: mitral peak velocity of late filling; E': early diastolic mitral annular velocity; A': late diastolic mitral annular velocity; BNP: B type natriuretic peptide. WKY: age-matched normotensive Wistar-Kyoto rats, *n* = 7; SHR-Baseline: 15-month-old spontaneously hypertensive rats, *n* = 7; SHR-C: 19-month-old spontaneously hypertensive rats received placebo for 18 weeks, *n* = 7; SHR-B: 19-month-old spontaneously hypertensive rats received BGP-15 for 18 weeks, *n* = 7. ^∗^*p* < 0.05 vs. WKY, ^∗∗^*p* < 0.01 vs. WKY, ^#^*p* < 0.05 vs. SHR-Baseline, ^##^*p* < 0.01 SHR-Baseline, ^§^*p* < 0.05 vs. SHR-C, ^§§^*p* < 0.01 vs. SHR-C.

## Data Availability

The authors confirm that all data is fully available without restriction. All relevant data is described within the paper.
